# pyIHM: Indirect Hard Modeling, in Python

**DOI:** 10.1021/acs.analchem.4c06484

**Published:** 2025-02-24

**Authors:** Francesco Bruno, Letizia Fiorucci, Alessia Vignoli, Klas Meyer, Michael Maiwald, Enrico Ravera

**Affiliations:** † CERM and Department of Chemistry “Ugo Schiff”, University of Florence, Via Luigi Sacconi 6, Sesto Fiorentino 50019, Italy; ‡ Consorzio Interuniversitario Risonanze Magnetiche di Metalloproteine, Via Luigi Sacconi 6, Sesto Fiorentino 50019, Italy; § Division Process Analytical Technology, Bundesanstalt für Materialforschung und -prüfung (BAM), Richard-Willstätter-Straße 11, 12489 Berlin, Germany; ∥ Florence Data Science, University of Florence, Viale G.B. Morgagni 59, Firenze 50134, Italy

## Abstract

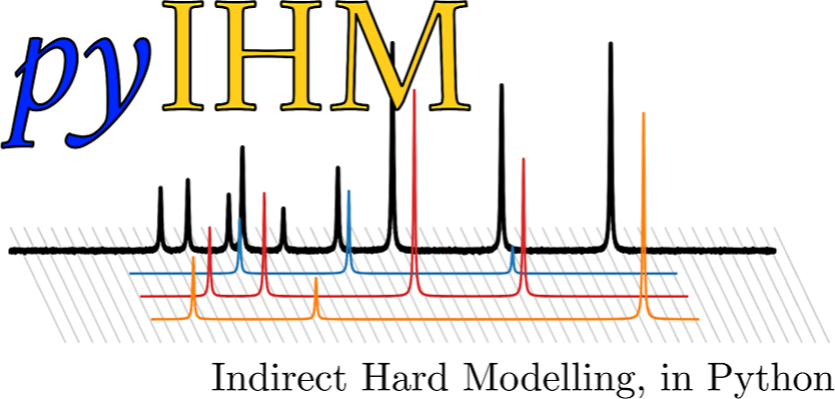

NMR is a powerful analytical technique that combines an exquisite qualitative power, related to the unicity of the spectra of each molecule in a mixture, with an intrinsic quantitativeness, related to the fact that the integral of each peak only depends on the number of nuclei (i.e., the amount of substance times the number of equivalent nuclei in the signal), regardless of the molecule. Signal integration is the most common approach in quantitative NMR but has several drawbacks (vide infra). An alternative is to use hard modeling of the peaks. In this paper, we present pyIHM, a Python package for the quantification of the components of NMR spectra through indirect hard modeling, and we discuss some numerical details of the implementation that make this approach robust and reliable.

## Introduction

Nuclear magnetic resonance is a well-established technology, which allows for detailed investigations of both the qualitative and the quantitative description of complex chemical and biological samples. Its analytical applications encompass food, drug discovery reaction mixtures, and other fluids of biological origin, to name a few. NMR is both qualitative, in that each molecule has its own spectra, different from the spectra of any other molecule, and quantitative because the intensity of each signal solely depends on the number of nuclei that contribute to it times the amount of substance, regardless of the environment in which the interrogated nucleus resides.^1^

NMR is often sensitivity-limited^2^ because of the small population difference between the nuclear spin states involved in the transitions. For quantitative NMR applications, however, sensitivity is usually not a limiting factor: most quantitative NMR experiments are acquired on ^1^H, which has the highest receptivity among all the magnetically active nuclei (the second-highest gyromagnetic ratio combined with almost 100% natural abundance^3^). Furthermore, concentrated solutions are usually analyzed. These aspects make the quality of the recorded spectra very high in terms of the signal-to-noise ratio. The LOD of NMR is on the order of micromolar for high field NMR to millimolar for benchtop instruments.

Since the very dawn of the technique, quantitative analysis of NMR spectra has been performed by integration of the characteristic signals of the molecules of interest. However, the integration of the spectra can be limited by the resolution of the spectra: to account for the 99% of the intensity of a Lorentzian signal, the integration has to be computed over a region spanning a frequency range that is 64 times the full-width at half-maximum of the signal.^4^

In practical applications, it is very uncommon to find a characteristic signal for each component of the mixture that fulfills this requirement. In addition, the integration of the signal suffers from baseline distortion and incorrect phasing. Phasing the spectrum aims to confine the absorption Lorentzian in the real part of the spectrum and the dispersion Lorentzian in its imaginary part. The presence of a dispersion component in the real part of the spectrum results in negative features around the peaks, which are subtracted from the intensity of the signal during the integration. Baseline distortions introduce the same kind of errors but can also include offsets from zero-baseline, which further jeopardize the correct computation of the signal intensity.

A rather obvious workaround when integration is not possible is to fit the signals of interest and extract their intensity accordingly. This approach is much less limited by the resolution of the data and is less sensitive to phase and baseline distortions, as these can be included in the model. However, a single NMR signal is described by (at least) four parameters (i.e., intensity, chemical shift, line width, and Lorentzian/Gaussian ratio); therefore, the complexity of the fitting procedure escalates with increasing number of components, if one tries to fit the spectrum as a whole. On the contrary, the task becomes rather simple if one assumes that the spectrum of a mixture is given by a convex combination of the spectra of its individual components. This procedure goes under the name of indirect hard modeling (IHM).^5^ Hard modeling means to build a “hard model” where every parameter has a physical meaning, as laid out in the previous paragraph, with the further assumption that the contribution of the spectra of each individual component makes the spectrum of a mixture. The beauty of IHM lies in not directly addressing the highly complex mixture spectra, but instead in building the individual hard models for each pure component based on separate, clean spectra. These component models then act as building blocks to predict the final mixture spectrum based on the concentration of each species. This approach offers several advantages. It provides robust quantification, even in the presence of severe peak overlap. In addition, with minimal modifications, IHM can account for intermolecular interactions that alter peak positions, a common issue in concentrated mixtures, or line shapes (across different instruments or in series of samples), as it is sufficient to allow for small adjustments of the peak features during the fit of the spectrum.^6^ Such a structure favors the performance of the optimization mechanisms.

While IHM requires some initial setup, in particular the creation of the component models, the payoff is significant. It offers the possibility to extract reliable quantitative information from complex NMR spectra, making it a valuable tool for analytical chemists working with mixture analysis.^7^ The use of IHM is indeed so obvious that it is implemented in commercial software options or routine “in house” scripts. Examples include PERCH (https://www.perchsolutions.com/),^8^ or MestreNova (https://mestrelab.com/),^9^ or S-PACT PEAXACT (https://www.s-pact.de/en),^10^ or the metabolomics-oriented Chenomx (https://www.chenomx.com/).^11^ Also, open-source alternatives based on similar approaches to IHM exist.^12−14^ Apart from the individual pros and cons of the different implementations (costs, long-term sustainability, and maintenance), it is important to remark that the setup of the initial spectral database is crucial not only for the success of the analysis, but also for the usability of the computational tool: analysts dealing with reaction monitoring in nonaqueous solvents do not care about large molecule databases obtained in water, but are more interested in very specific collections that represent the actual possible outcome of the reaction they are optimizing.

There might be cases where a pure component model is not accessible because it is an unstable short-living intermediate that cannot be isolated from a complex mixture. With methods like complemental hard modeling it is possible to generate a single unidentified component model from an otherwise completely known mixture model based on residual fitting. Hard modeling factor analysis identifies all pure components in an unknown chemical system by using multivariate statistics.^15^ Another possibility is the use of prior knowledge to generate the quantum mechanical (QM) models. Even a complex mixture NMR spectrum consisting of thousands of individual signals can be described by QM theory.^16^ This has a long history in structural analysis^17^ and strongly benefits from the increased quality^18^ and availability of QC methods^19^ of efficient software tools for NMR spectra calculations,^20,21^ and of computing power, allowing for calculation of spectra within seconds. A large field of application is pharmaceutical identity testing and quality control^22^ because the high-level of automation makes these methods especially interesting in high-throughput screening and routine operations. Software packages as Cosmic Truth (https://www.nmrsolutions.fi/), ChemAdder (https://www.chemadder.com/), and USP-ID (https://mestrelab.com/software/usp-id/) make it more accessible and easier-to-use to NMR scientists. Some of them already include databases that can be individually extended with user-specific data sets.

While the initial developments of QM spectral analysis (QMSA) techniques were aimed for structural identification and deconvolution of complex mixture spectra, the quantitative use (qQMSA) of it by fitting individual QMSA pure components has been demonstrated in several applications. This shows also very promising results when applied to spectra at lower dispersion, as typically obtained from compact benchtop NMR instruments, which become more popular in reaction monitoring and PAT applications due to their portable use.^23,24^

## Experimental Section

Benzoic acid (BzAc) was provided as the primary calibrator NIST PS1 from National Institute for Standards and Technology (NIST, Gaithersburg, USA). Ethylene carbonate (EC) was purchased as an analytical standard (HPC Standards GmbH, Borsdorf, Germany). Ochratoxin-A and 1,2,4,5-tetrachloro-3-nitrobenzene (TCNB) were both purchased in high-purity forms, the latter from TraceCERT product line for qNMR (Sigma-Aldrich Chemie GmbH, Taufkirchen, Germany). All gravimetric operations were performed on an ultramicrobalance (XP2 U/M, Mettler-Toledo, Gieβen, Germany). After separate weighing on aluminum weighing dishes, the materials were combined and completely dissolved in appropriate amount of deuterated solvent (Deutero AG, Kastellaun, Germany). Solution was transferred into a 5 mm NMR tube for measurement.

NMR experiments were performed on a 500 MHz NMR spectrometer system (VNMRS500, Varian Associates, Palo Alto, USA) operating at a proton frequency of 499.9 MHz, which was equipped with a 5 mm OneNMR probe. Acquisition was performed with accumulation of 16 to 32 scans. Relaxation delay was set to exceed seven times *T*_1_ relaxation time to ensure quantitative response from all nuclei. *T*_1_ relaxation times were either estimated by a preliminary inversion–recovery experiment or from certificate information on the used internal standard. The spectrum of mock urine was taken from ref (25).

The peak integration for the comparison of the results obtained with pyIHM was performed using the function processing.integrate of the KLASSEZ package. The integration regions were selected by fulfilling the rule of 64 times the line width of the signals. The aromatic region of benzoic acid was addressed by integrating the three signature multiplets together and making their sum count as five protons.

The pyIHM package is freely available for download on GitHub and PyPI at the following links: https://github.com/MetallerTM/pyihm, https://pypi.org/project/pyihm/. Table S1 contains the additional packages required to install and operate the software. The optimization algorithm mentioned throughout the main text and employed by pyIHM is implemented “as is” in the *lmfit* python package. All the target functions, instead, are written by us in python. Further details on the implementation are discussed in the user manual.

### Theory

IHM analyses start with the creation of hard models for all of the components that are assumed to be present in the mixture. The hard model dictated by the physics of the FT-NMR experiment is an oscillating and exponentially decaying complex signal in the time domain, which is Fourier transformed to obtain a Lorentzian line shape.^26^ For this reason, it is natural to build a hard model in the time domain. This choice comes with additional benefits. The assumption that the field is perfectly homogeneous throughout the sample is often not fulfilled: the simplest approximation of the field inhomogeneity is through a Gaussian distribution of frequencies convoluted to the signal in the frequency domain, which corresponds to a multiplication of the time domain signal by a Gaussian.^27−29^ To compensate for slight distortions of the pure Lorentzian shape arising from different origins, without the expectation of addressing their physical meaning, we use a different fraction of Gaussian for the different signals (i.e., the Voigt shape). The model signals are thus simulated in the time domain according to the equation
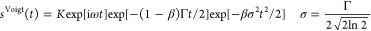
1The parameters that describe each signal are the relative intensity *K*, the frequency with respect to the carrier ν = ω/2π (which will then be translated in the chemical shift δ), the full-width at half-maximum Γ, and the fraction of Gaussian β (β = 0 for pure Lorentzian, β = 1 for pure Gaussian). A visual interpretation of the parameters is given in Figure S1. In this paper, we will assume that the phase and the baseline distortions can be easily corrected either automatically or manually (which is usually the case in the majority of industrial reaction monitoring applications). These issues will be addressed in future work, including more sophisticated models of instrumental fingerprint.^30^

The experimental spectrum *S*^exp^ is described as the sum of the *M* component spectra resulting from *M* chemical species. Under the assumptions listed above, our model function *f* is given by
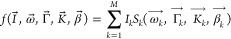
2where  is the spectrum of the *k*-th pure component, obtained by the Fourier transform of the sum of *N*_*k*_ Voigt line shapes (eq 1)
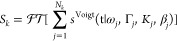
3

The cost function of pyIHM is to find the optimal set of parameters  that minimize the difference between the experimental and simulated spectra (eq 4) in the least-squares sense
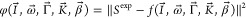
4

It is possible to restrict the search to those areas that contain the signals of interest, to reduce the computational burden, as well as to exclude nonrelevant signals.

If the signals of interest are too close to nonrelevant peaks to trim them out, dummy peaks can be added as an extra component to take nonassigned features into account.

## Results and Discussion

### pyIHM Implementation

#### Initial Guess

The peak parameters for the computation of the pure components spectra are read by pyIHM from a “.fvf” file, which is generated by dedicated functions in KLASSEZ (Figures S2–S4).^31^ An example script on how to create a.fvf file is given in the Supporting Information. The origin of the parameters can be from the experimental spectra of the individual components, or using the knowledge gathered from previous analyses, or from literature, or from calculations.^32−34^ The graphical user interfaces (GUIs) and their functioning are explained in detail in the pyIHM manual, Section 2.1.

Once loaded in pyIHM, the initial guess can be further refined by employing two back-to-back GUIs. The first GUI (Figure S5a) allows for calibration and coarse correction of the chemical shifts to account for calibration and field drifts, as well as to assign the initial concentrations to the components of the mixture. By clicking on the “EDIT” button in this interface, the second GUI appears (Figure S5b). Here, the spectrum of only one component is broken down into its individual peaks, which can be finely adjusted in a similar fashion, as shown in Figure S2. The process can be repeated as many times as needed, until the “SAVE” button in the first GUI is pressed.

#### pyIHM Core Fit

In the computation of model function *f* of eq 3, singlets and multiplets are treated differently. When dealing with multiplets, the shifts of the features included in the fine structure of the peaks are defined as differences with respect to a central shift (corresponding to the center of the multiplet). The use of individual peaks for modeling multiplets, rather than a model based on the calculation of their relative intensities based on the spin Hamiltonian allows us to mitigate the computational cost and to reduce the covariance between peak positions and intensities, which sizeably complicates the performance of fitting algorithms.

#### Optimization of the Chemical Shifts

**Figure 1:**
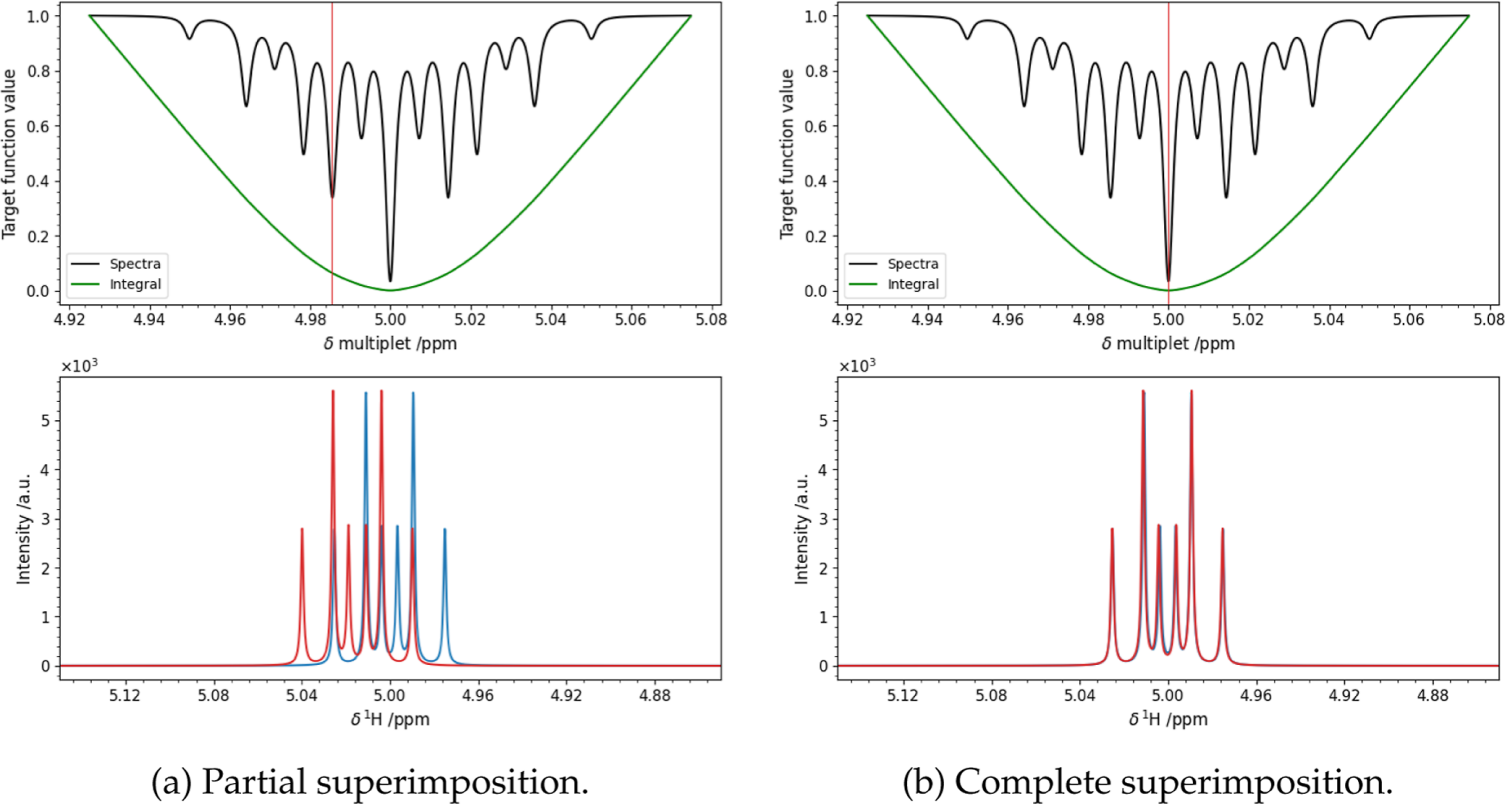
Signal centered at 5 ppm was simulated at 700 MHz ^1^H Larmor frequency as a doublet of triplets with scalar coupling constants *J* = 15 and 10 Hz (blue trace, bottom panels). All the six features of the multiplet have the same line width of 1 Hz. The model signal (red trace, bottom panels) was simulated with the same parameter, and its chemical shift is varied from 4.975 to 5.075 ppm in steps of 6.10 × 10^–4^ ppm. For each of these values, the value of the target function was computed on the difference of the spectra (eq 4, black trace in the top panels) and of their integrals (eq 5, green trace in the top panels). Here are shown two particular values of chemical shift, marked with a red line on the top panels: one correspondent to partial superimposition of the features (a) and the true value (b). The figure shows that a partial superimposition between experimental and model spectrum does not correspond to a secondary minimum of the integral target function.

One unique feature of pyIHM is in the fit of the chemical shifts to get the correct peak positions. The approach that we report here serves the scope of significantly increasing the efficiency of the fit. To illustrate the numerical issues associated with this part of the fit, let us suppose that we know exactly the structure of a given multiplet and the relative intensities of the fine structure of the multiplet, but that we do not know its chemical shift. In this case, the optimization of our target function (eq 4) would require changing a single parameter (that is, the chemical shift δ). In this scenario, we can draw the complete error surface across the values of δ (black trace in the top panels of Figure 1). From this graph, it is apparent that the numerical problem is that a little misalignment between the experimental and calculated signal translates in local minima of the target function, which will create problems for all nonlocal algorithms. Depending on the fine structure of the signal, the actual shape of the error surface may change but its roughness remains a common feature.

To find the correct alignment, instead of going for global optimizers, which are slower than the local ones, we selected a target function that gives rise to a smoother error surface, as proposed earlier for EPR spectroscopy.^35−37^ A convenient choice is the target function φ^algn^(ω) computed as the difference between the integral (i.e., the cumulative sum) of the experimental and calculated spectrum (eq 5), depicted in this case as green trace in Figure 1.
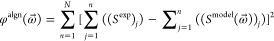
5

Of note, the alignment fit with this latter target function is insensitive to line width overestimates (see Figure S6).

To summarize, the fitting procedure goes through the following steps:

1 The guess model of the regions of interest is created as described above (Figure S7);
2 The peaks of the guess are thus aligned by adjusting the chemical shift based on the integral (Figure 1, eq 5) using the Levenberg–Marquardt least-squares algorithm;
3 All the parameters of the model are fitted, using the difference of the spectra as target function (ref eq 4). The user can select customized fitting settings, or choose among two default modes: “fast”, that corresponds to a single optimization with Levenberg–Marquardt algorithm, and “tight”, consisting of a Nelder–Mead optimization followed by another one, performed with Levenberg–Marquardt algorithm.

In step #3, the “tight” option might be preferred over the “fast” one because of the different behavior of the two optimization algorithms. Levenberg–Marquardt is better suited for refinement of the fit results near the optimum as it falls toward the nearest minimum. Instead, a prior optimization step with the Nelder–Mead simplex is able to drive the parameters closer to the global optimum, hence improving the overall result of the fit.^38^

### Applications

The examples of application of pyIHM reported in this section are very simple and well-resolved spectra. This choice is justified by the need for reference data to prove the reliability of the program, which necessarily comes from the peak integration approach. It is therefore needed that the resolution of the signals fulfills the requirements for quantitatively accurate analysis. In this section, the results were obtained using the “tight” option in step #3 of the optimization. For comparison, the data computed with the “fast” option are shown in Supporting Information.

**Figure 2:**
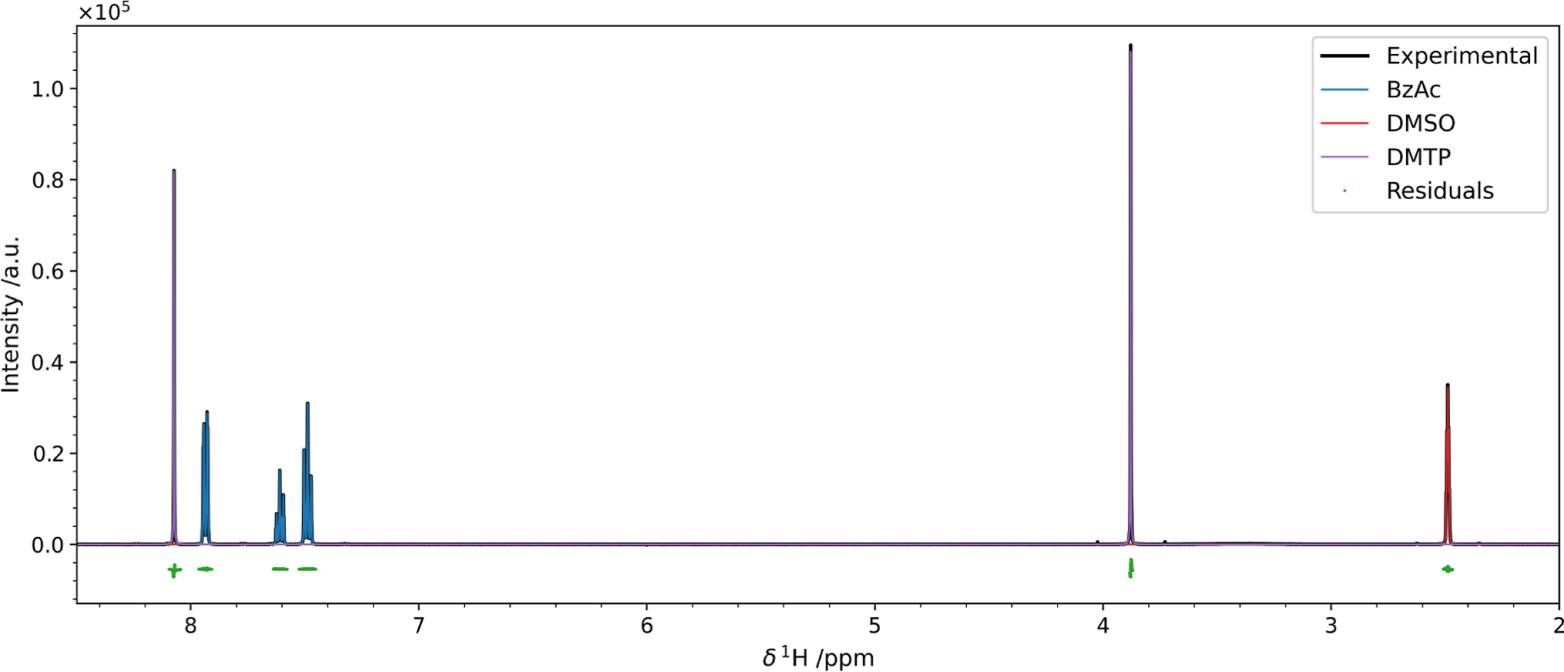
Result on the pyIHM deconvolution performed on a mixture of benzoic acid (blue) and DMTP (orange) in DMSO-*d*_6_ (red). The residuals of the fit are shown in green with a slight offset. Details on their statistical properties are given in Figure S8. The intensities were scaled to the integral of the total model trace for normalization purposes. Table 1 compares the obtained composition with the one obtained by peak integration.

As a first result, we present the fit of a mixture of two internal standards commonly used in quantitative NMR, benzoic acid (BzAc), and dimethylterephthalate (DMTP) in deuterated dimethyl sulfoxide (DMSO-*d*_6_). In this example, the positions of the initial guess were perfectly superimposed with the experimental spectrum, leaving the concentrations as the only parameters to be determined. Figure 2 and Table 1 show the results of this fit. The deconvolution is very good as the fit residuals are not polarized and tightly clustered around the mean (Figure S8), and the intensities are in line with the ones obtained with the traditional integration approach. It should be noted that since it is deuterated, the actual amount of DMSO in the mixture will not be accurate. However, this is not relevant for our analysis because we are comparing the pyIHM results with the integrals of the spectra, which bear the same information.

**Table 1:** Composition of the Mixture of Benzoic Acid and Dimethylterephtalate in DMSO-*d*_6_ Computed with pyIHM, Compared with the Intensities Obtained by Peak Integration

component	from pyIHM (%)	from integr (%)	diff (%)
BzAc	59.82	60.24	0.42
DMSO	19.82	19.89	0.07
DMTP	20.36	19.87	0.49

**Figure 3:**
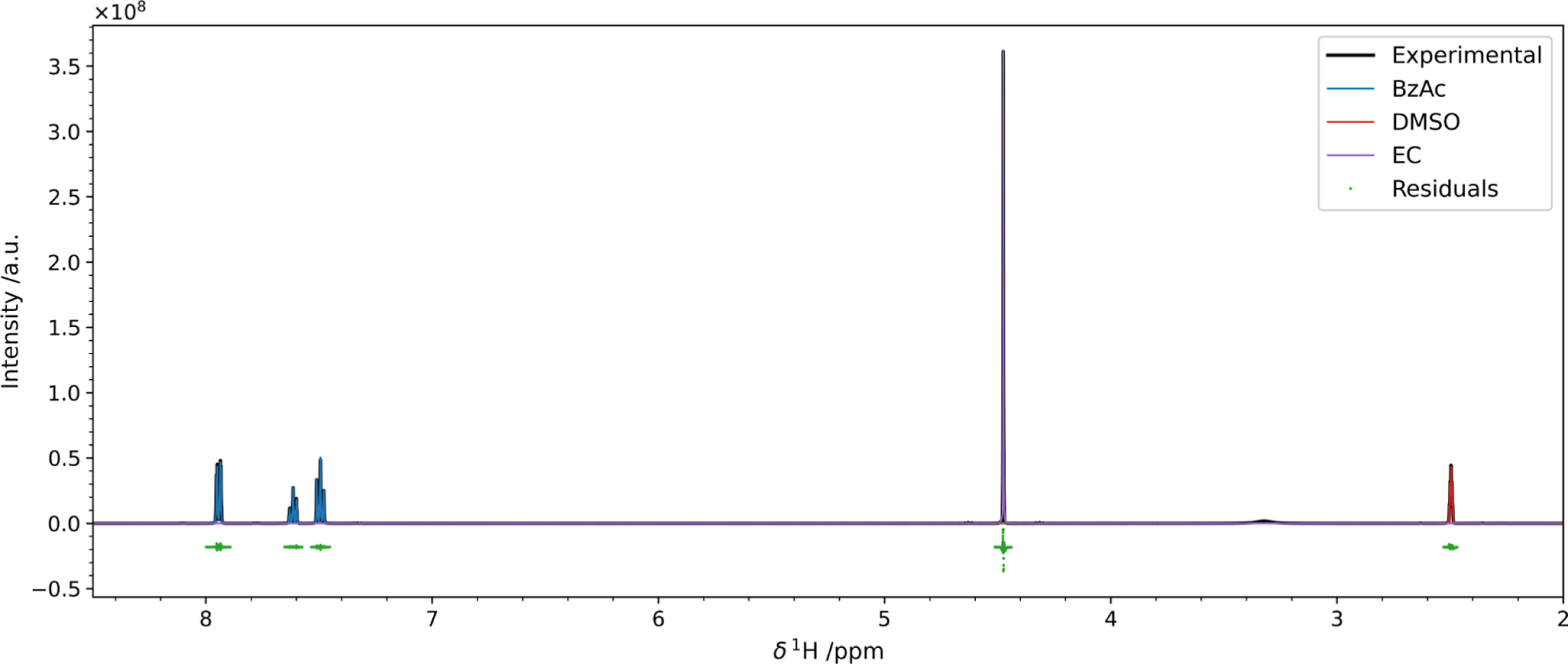
Results of the pyIHM deconvolution performed on a mixture of benzoic acid (blue) and ethylene carbonate (orange) in DMSO-*d*_6_ (red). The residuals of the fit are shown in green with a slight offset. Details on their statistical properties are given in Figure S11. The intensities were scaled to the integral of the total model trace for normalization purposes. Table 2 compares the obtained composition with the one obtained by peak integration.

To prove the power of the chemical shift optimization described in the previous section, we analyzed a mixture of two different internal standard than in the previous test, benzoic acid and ethylene carbonate (EC) in DMSO-*d*_6_, whose chemical shifts were not aligned with the experimental signals. As shown in Figure 3 and Table 2, also in this case the results are comparable with the integrated intensities.

**Table 2:** Composition of the Mixture of Benzoic Acid and Ethylene Carbonate in DMSO-*d*_6_ Computed with pyIHM, Compared with the Intensities Obtained by Peak Integration

component	from pyIHM (%)	from integr (%)	diff (%)
BzAc	49.47	50.24	0.77
DMSO	12.96	12.75	0.21
EC	37.57	37.01	0.56

**Figure 4:**
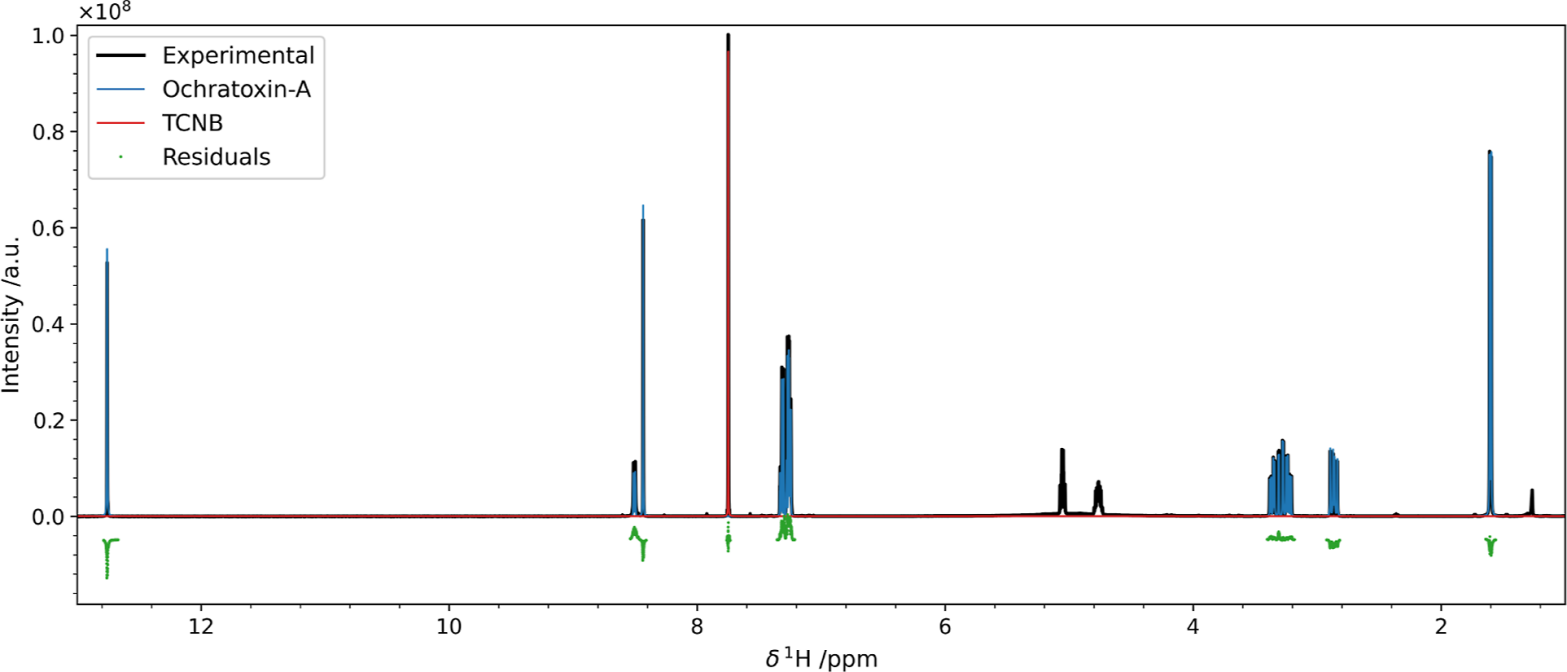
Results of the pyIHM deconvolution performed on a mixture of ochratoxin-a (blue) and TCNB (red). The residuals of the fit are shown in green with a slight offset. Details on their statistical properties are given in Figure S14. The intensities were scaled to the integral of the total model trace for normalization purposes.

A specific branch of mixture quantification is the purity check of a given compound. In this case, the mixture is composed of known amounts of analyte (ochratoxin-a) and an internal standard (tetrachloronitrobenzene, TCNB) (Figure 4). To this end, we weighted *m*_A_ = 6.8033 mg of analyte sample and *m*_S_ = 4.8674 mg of 99.79% pure internal standard, which correspond to *n*_A_ = κ × 16.848 mmol ochratoxin-a and *n*_S_ = 18.618 mmol TCNB. The theoretical ratio between the two components is 0.90493. The relative concentrations given by pyIHM are *x*_A_^f^ = 0.4684 and *x*_S_^f^ = 0.5316, with an intensity ratio of 0.8811. The purity κ of ochratoxin-a is then simply the ratio between the observed intensity ratio and the theoretical intensity ratio κ = 0.8811/0.90493 = 0.9737. The obtained value of 0.9737 is comparable to the value obtained with the classical integration analysis, which is 0.983, thus proving the reliability of the approach.

**Figure 5:**
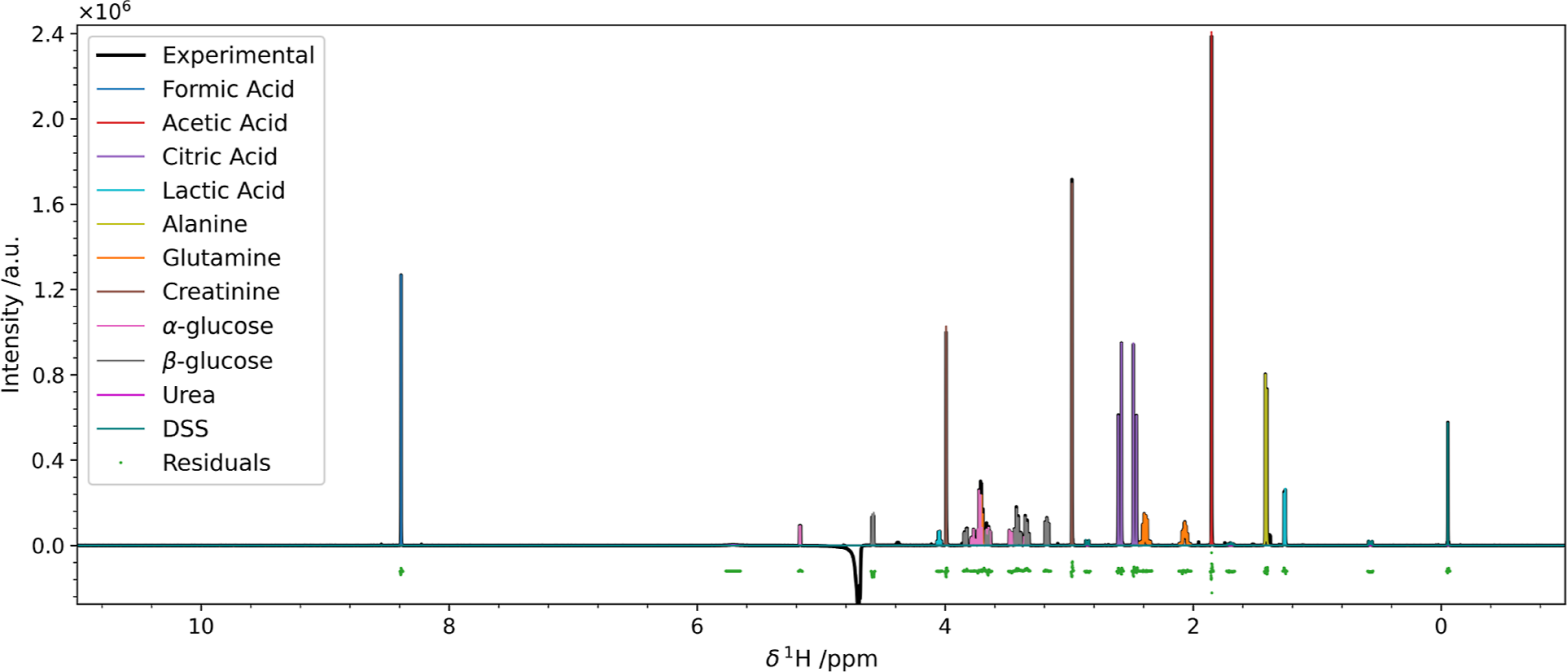
Results of the pyIHM deconvolution of the synthetic urine spectrum. The obtained concentrations are listed in Table 3.

As a final example, we prove that we can quantify more complex mixtures and to do so with higher accuracy than standard software. We used a mock urine sample composed of 10 metabolites at an approximate concentration of 10 mmol dm^–3^ and DSS as an internal standard at the concentration of about 1 mmol dm^–3^. The spectra were taken from.^25^ The result of the pyIHM deconvolution is shown in Figure 5. For this fit, we used the “custom” option with one Nelder–Mead simplex optimization followed by two Levenberg-Marquardt runs, for increased accuracy. A closer look at the specific regions is reported in Figure S18.

The quantitation was also performed using the Chenomx NMR suite 11.0. The obtained composition with both pyIHM and Chenomx is reported in Table 3.

**Table 3:** Relative Concentrations of the Synthetic Urine Spectrum Obtained by pyIHM, Compared with the One Obtained by Chenomx^a^

component	rel. conc		rel. DSS	
	pyIHM (%)	Chenomx (%)	pyIHM	Chenomx
formic acid	16.658	22.994	13.72	31.7
acetic acid	13.919	14.451	11.47	19.9
citrate	15.717	17.858	12.95	24.6
lactate	3.128	2.252	2.577	3.10
alanine	9.634	10.380	7.936	14.3
glutamine	12.05	9.723	9.929	13.4
creatinine	13.79	11.175	11.37	15.4
α-glucose	6.045		4.979	
β-glucose	7.063		5.818	
glucose total	13.108	8.237	10.80	11.3
urea	0.768	2.203	0.633	3.03
DSS	1.214	0.726	1.000	1.00

a The quantification of urea by NMR is known to be very inaccurate.

Although the fit seems to be acceptable, the intensity of formate with respect to DSS was estimated to be about 31, which is very distant from both the 13.7 given by pyIHM and the expected value of about 10. An explanation of such high inaccuracy can be identified in the incorrect line width estimation, which is not an adjustable parameter in Chenomx. Additionally, we can quantify both glucose species, and the ratio among the two is 46/54. The discrepancy from the expected ratio of 34/66 can be explained considering that the resonance of the β anomeric proton is very close to the solvent signal; hence, its intensity can be hampered by the solvent suppression by about 12%.

## Conclusions

In this article, we describe pyIHM, a user-friendly python package for quantification through 1D NMR spectra, based on the IHM approach, which represents a valid alternative to traditional peak integration. IHM is less affected by the resolution of the experimental spectrum, and the use of multiple signals at once for the quantitation improves its robustness.

pyIHM has a few unique features. The generation of the hard model is made easy both via a GUI-assisted deconvolution of the experimental spectra of the pure component of the mixture or from simulated data. Both options are provided by the KLASSEZ package through the use of simple python scripts.

The other feature is that we reserve a special treatment for the optimization of the chemical shifts of multiplets. We demonstrate how the use of a target function computed on the integrals of the spectra leads to the fitting of the peak positions to the global optimum, thus ignoring the secondary minima arising from partial superimposition of the multiplet fine structure components.

Finally, generation of the model in the time domain allows for a simpler handling of the experimental fingerprint.

This software package is openly available through GitHub, together with detailed documentation.

## Supporting Information Available

Requirements of the pyIHM package; instructions for generating the initial guess using KLASSEZ and example scripts; description of the pyIHM workflow; and additional data about pyIHM quantification results (PDF)
