# Supplementary material for: pyIHM: Indirect Hard Modeling, in Python
Source: Anal Chem. 2025 Feb 24;97(8):4598–605. doi: 10.1021/acs.analchem.4c06484 (PMC11883739; doi:10.1021/acs.analchem.4c06484)
Supplement: Supplementary file 1 — ac4c06484_si_001.pdf [file ac4c06484_si_001.pdf]

Supporting Information: pyIHM: Indirect Hard Modelling, in Python

Francesco Bruno,*,†,‡ Letizia Fiorucci,†,‡,⊥ Alessia Vignoli,†,‡ Klas Meyer,¶ Michael Maiwald,¶,§ and Enrico Ravera*,†,‡,‖

†*CERM and Department of Chemistry "Ugo Schiff", University of Florence, Via Luigi Sacconi 6, Sesto Fiorentino, 50019, Italy*

‡*Consorzio Interuniversitario Risonanze Magnetiche di Metalloproteine, Via Luigi Sacconi 6, Sesto Fiorentino, 50019, Italy*

¶*Bundesanstalt für Materialforschung und -prüfung (BAM), Division Process Analytical Technology, Richard-Willstätter-Straße 11, 12489 Berlin, Germany*

§*Deceased, August 16th, 2023*

‖*Florence Data Science, University of Florence, Viale G.B. Morgagni 59, Firenze, 50134, Italy*

⊥*Present Address: Max Plank Institut für Kohlenforshung, Kaiser-Wilhelm-Platz 1, 45470, Mülheim an der Ruhr, Germany*

E-mail: bruno@cerm.unifi.it;francesco.bruno@unifi.it; ravera@cerm.unifi.it;enrico.ravera@unifi.it

Contents

1 pyIHM requirements **S-2**

2 Generation of the initial guess using KLASSEZ **S-2**

2.1 Example scripts S-6

3 Insights on pyIHM workflow S-13

4 Additional data about pyIHM results S-15

1 pyIHM requirements

Table S1: Additional package requirements for pyIHM.

Package	Version
Python	$\geq$ 3.9
Klassez	$\geq$ 0.4a.7
Numpy	$\geq$ 1.22.0
Scipy	$\geq$ 1.11.2
Matplotlib	$\geq$ 3.8
Seaborn	$\geq$ 0.13.2
Lmfit	$\geq$ 1.2.2
Csaps	$\geq$ 1.1.0
Nmrglue	$\geq$ 0.9
Jeol-parser	$\geq$ 0.1.2

2 Generation of the initial guess using KLASSEZ

The procedure for extracting the parameters from the experimental spectra of the individual components consists of three steps. First, the experimental spectrum is imported, either as an FID or as already processed data. In the former case, the processing will be performed internally to the script. Then, the initial guess for the fit is generated interactively, through the dedicated user interface shown in figure S2. The user can place as many Voigtian components as necessary, varying their positions, linewidths, lineshapes, and relative intensities, according to equation 1 (visually represented in figure S1). It is possible to work on more restricted regions of the spectrum, to decrease the computational workload. Although this is the most accurate way to generate an initial guess, this manual process can be tedious if the spectrum features many sharp peaks. Therefore, we implemented an

alternative interface, that employs a peak-picker to estimate the peak positions and their linewidths. The intensity is inferred by integration of a restricted region around the center of the peak. The user can interactively control the detection parameters of the peak-picker (i.e. the threshold and the prominence) and the extent of the integration window, and they can also add or remove peaks manually. An example of this interface is shown in figure S3. Finally, the initial guess is refined by minimizing the difference from the experimental data in the least-squares sense, one region at the time. The output of this fit is a collection of all the parameters needed to simulate the deconvoluted spectra, which are collected in the ".fvf" file.

When one wants to use previous knowledge instead of experimental data, pyIHM relies upon the class "`Spectrum_1D`" in KLASSEZ. This reads a simulation input file, which consists of two parts. The first section contains the basic information of the virtual spectrometer, i.e. the strength of the magnetic field B_0, the observed nucleus, the number of points of the FID, the spectral width in ppm, and the position of the carrier in ppm. Then, a table of signal parameters follows. The user must specify the chemical shifts in ppm, the linewidths in Hz, the intensities, and the fractions of gaussianity of the signals to simulate. The relative intensities of the signals should be set to the number of nuclei that each signal accounts for. Furthermore, each signal can be splitted according to its fine structure by stating the expected multiplet type and the scalar coupling constants. Since the linewidths and the fraction of gaussianity are often not reported in spectral assignments, we have simulated the signals with $\Gamma = 2\text{-}5\,\text{Hz}$ and $\beta = 0$ (i.e. pure Lorentzian). Once the input file is loaded, the parameters contained in the input file are rearranged with a specific function to resemble the structure of a ".fvf" file, which is then saved. An example input file and the script to read it and generate the related ".fvf" file, are given in SI.

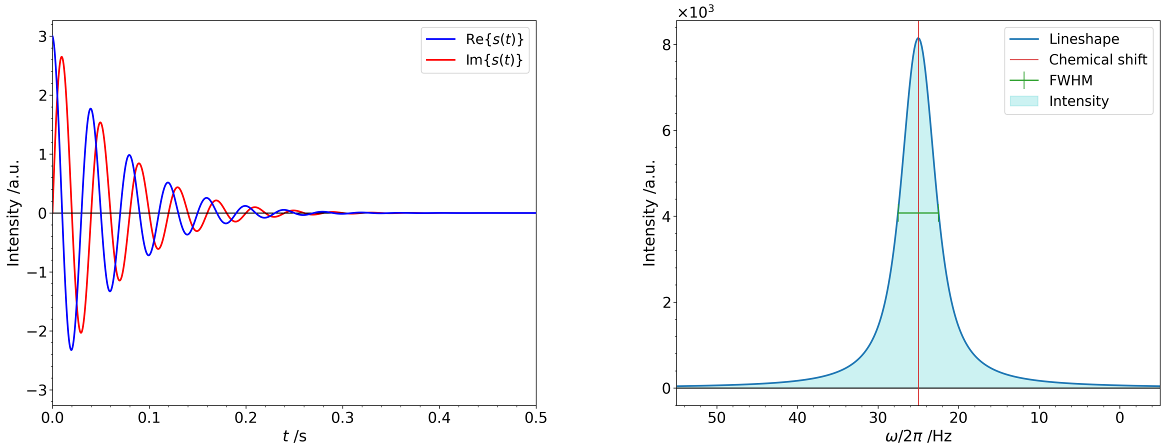

(a) FID of the signal.

(b) Fourier-transformed FID.

Figure S1: Voigt model generated according to equation 1, (a) in the time domain and (b) in the frequency domain (only real part displayed). The used parameters were: $K = 3, \nu = 25\,\text{Hz}, \Gamma/2\pi = 5\,\text{Hz}, \beta = 0.2$.

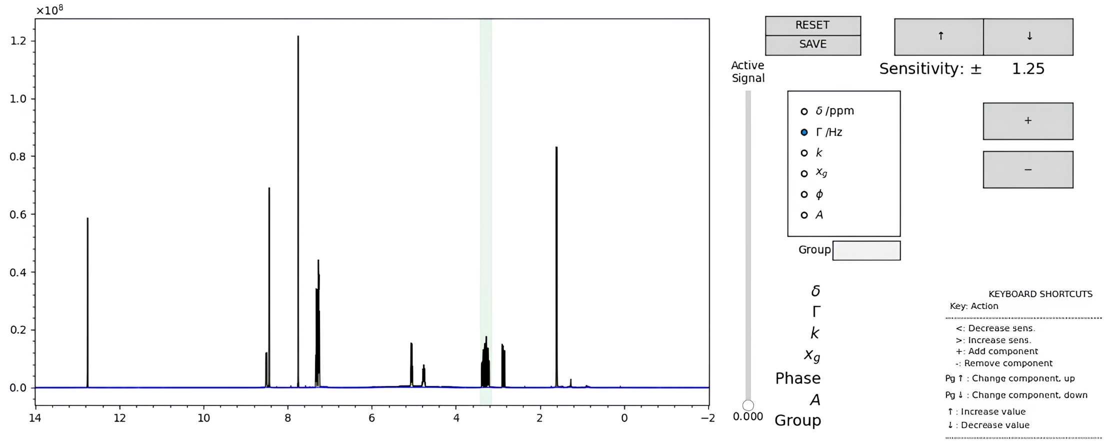

(a) Panel for the interactive selection of the regions of the spectrum to deconvolve.

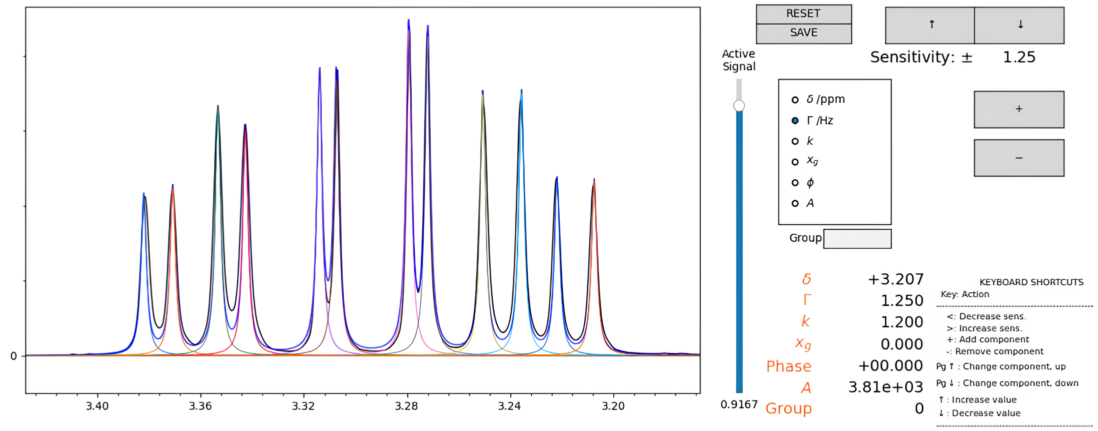

(b) Interface for the computation of the initial guess.

Figure S2: Example of the graphical interface for the generation of the guess. It is possible to focus on portions of spectra at once (panel *a*): the limits of the enlarged window (panel *b*) serve as boundaries for the fitting region. The user can add as many components as needed, and visually adjust their parameters according to equation 1 in order to match the appearance of the experimental spectrum. The "group" attribute marks feature of a same multiplet: group 0 (default option) denotes singlets. When the "save" button is pressed, the parameters are saved in a ".ivf" file, and a green span appears on the figure to mark the already processed regions. The process can be repeated until the interface is closed.

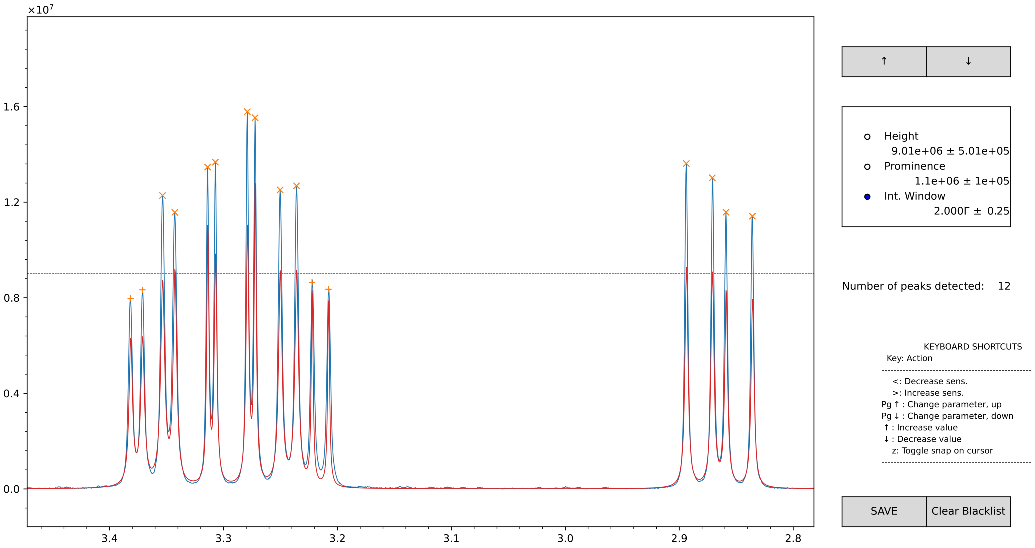

Figure S3: Example of the graphical user interface for the generation of the guess. As in figure S2a, it is possible to focus on restricted portion of the spectrum at once. The user can use the mouse scroll to move the threshold or prominence employed by the peak-picker for the detection of the signals. The detected positions are marked with orange $\times$, and the model (red trace) is automatically computed by estimating the linewidths and the integrals. In case the linewidth estimation would fail for some reason, a default FWHM of 5 Hz is set. The user can also manually add peak positions that are not detected automatically, which in this case would appear as orange $+$, or remove a peak that is automatically detected, by double-clicking with the left or right button of the mouse respectively. When the "**SAVE**" button is clicked, the active region is marked with a green span, and the initial guess of that region is written as a section of the *.ivf* file.

2.1 Example scripts

The deconvolution of an experimental spectrum can be performed by using the following script, replacing the `path` variable with the location of the dataset.

```
#! /usr/bin/env python3

import klassez as kz

## READ THE DATASET
# Path to the spectrum
```

```
path = 'test_spectrum.fid'

# spect='bruker', 'varian', 'magritek', 'oxford'
s = Spectrum_1D(path, spect='varian')

## PROCESSING
#    Window function
s.procs['wf']['mode'] = 'em'     # Exponential modulation
s.procs['wf']['lb'] = 0.2        # 0.2 Hz
#    Zero-filling
s.procs['zf'] = 2**16            # Up to 64k points

s.process()          # Apply processing and Fourier transform
s.adjph()            # Correct the phase, interactively

## FIT
filename = 'test'    # Base name for files and figures

# Looks for a file called '<filename>.ivf':
# if there is, loads it, if there is not, creates an empty one
# and opens the panel for interactive computation of the guess
s.F.iguess(filename=filename)

# Start the fit and save the results in '<filename>.fvf'
s.F.dofit(filename=filename,
        u_lim=1,              # Boundaries for chemical shift /ppm
        f_lim=10,             # Boundaries for linewidths /Hz
```

```
    vary_phase=False,    # Allow to change the phase
    vary_b=True,         # Allow to change fraction of
        gaussianity
    itermax=10000,       # Max. number of iterations
    fit_tol=1e-5,        # Arrest criterion
    )

# Save a series of figures of the output, in png, with the
    residuals
s.F.plot(filename=filename, show_res=True, res_offset=0.1, ext='
    png')
```

Alternatively, the spectrum can be simulated using an input file and a script. We present the simulation of the spectrum of alanine as template. The resulting spectrum is shown in figure S4.

Input file for spectra simulation:

```
### Instrument setup
B0        14.1                     # Magnetic field /T
nuc       1H                       # Observed nucleus
o1p       4.7                      # Carrier position /ppm
SWp       30                       # Spectral width /ppm
TD        2**17                    # Number of complex points of the
    FID

### Peak parameters
shifts    3.77, 1.47      # Chemical shifts /ppm, comma-separated
    values
fwhm      1, 1            # FWHM /Hz, comma-separated values
amplitudes        1, 3    # Relative intensities
b                 0, 0    # Fraction of gaussianity (0=lorentzian,
    1=gaussian)
# Multiplet type: s, d, t, q, dt,...
mult      q, d,
# Scalar coupling constants /Hz
Jconst    6, 6            # For multiple splitting: [J1, J2]
```

Python script for reading it:

```
#! /usr/bin/env python3

import klassez as kz

# Path to the input file
path = 'alanine.acqus'
# Read the file and make the FID
s = kz.Spectrum_1D(path, isexp=False)
# Generate the .fvf file. Extension added automatically.
s.to_vf(filename='alanine')
```

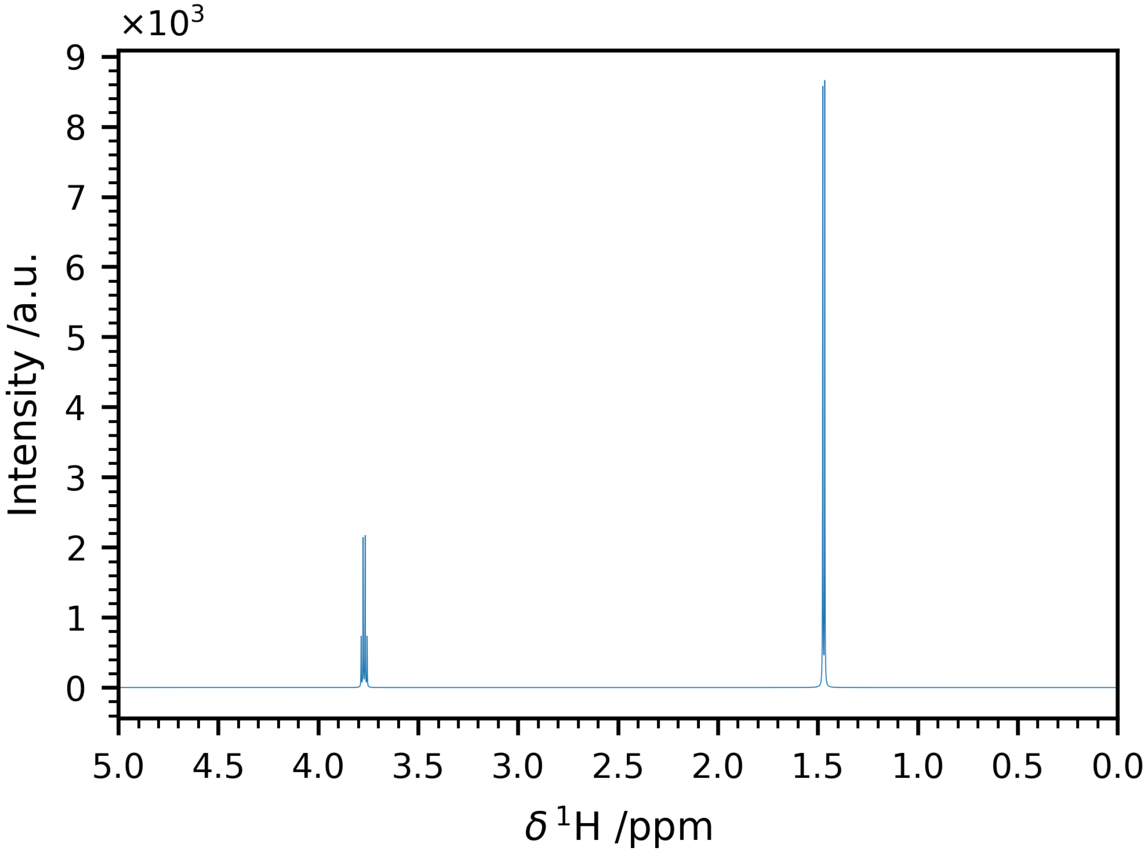

Figure S4: Simulated spectrum of alanine.

3 Insights on pyIHM workflow

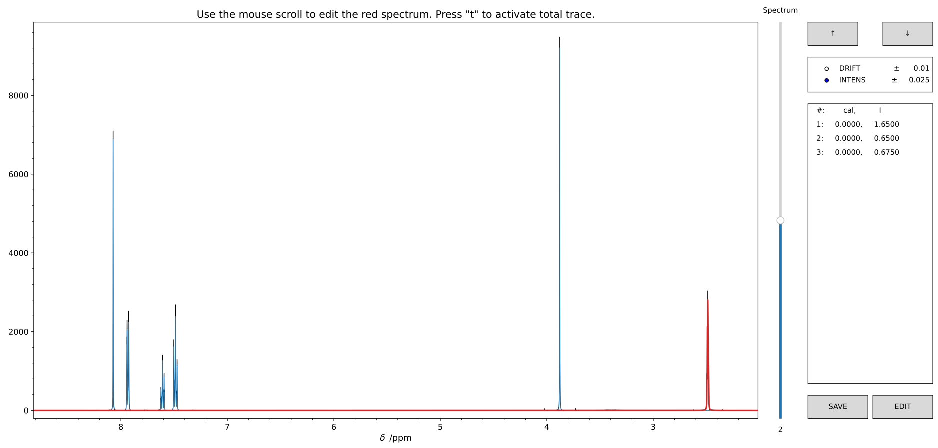

(a)

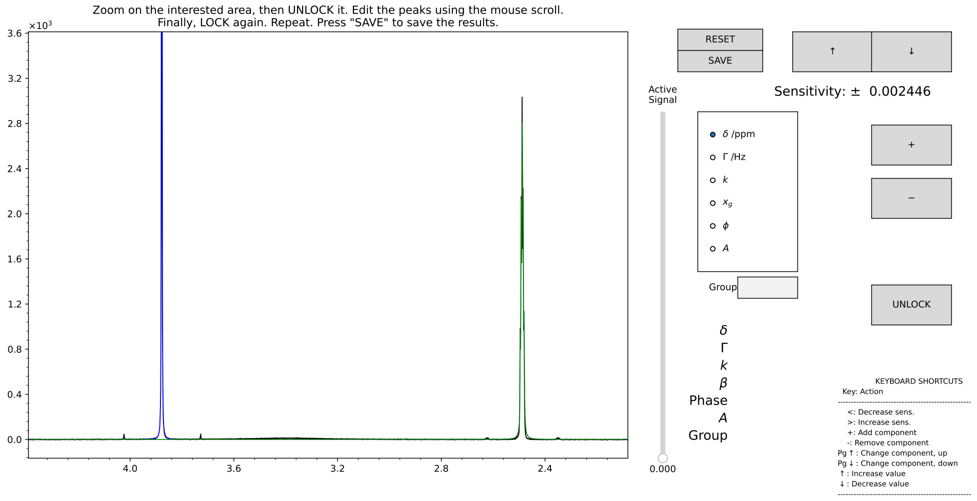

(b)

Figure S5: The refinement of the initial guess in pyIHM is performed by two GUIs that work back-to-back, synergistically. The first GUI, in panel **a**, allows for the selection of the concentration of the spectra, and to correct for drifts that affect the component as a whole. The user can change the active spectrum by moving the vertical slider. When the "EDIT" button is clicked, this GUI closes, and the one in panel **b** opens. In this interface, the user can edit the initial guess of the active spectrum peak-by-peak, in a similar fashion as for the manual computation of *.ivf* files (figure S2b). To decrease the computational workload that sits behind this interface, the active spectrum appears as a green trace, whereas the other components are shown in blue. The experimental mixture spectrum is drawn in black. The user has to focus on a particular window, and then press "UNLOCK": the visible region of the green spectrum will then unpack in editable components. Clicking "SAVE" on this GUI closes the interface, and the GUI in panel **a** opens up again. The process can then continue, until the "SAVE" button in the first GUI is pressed.

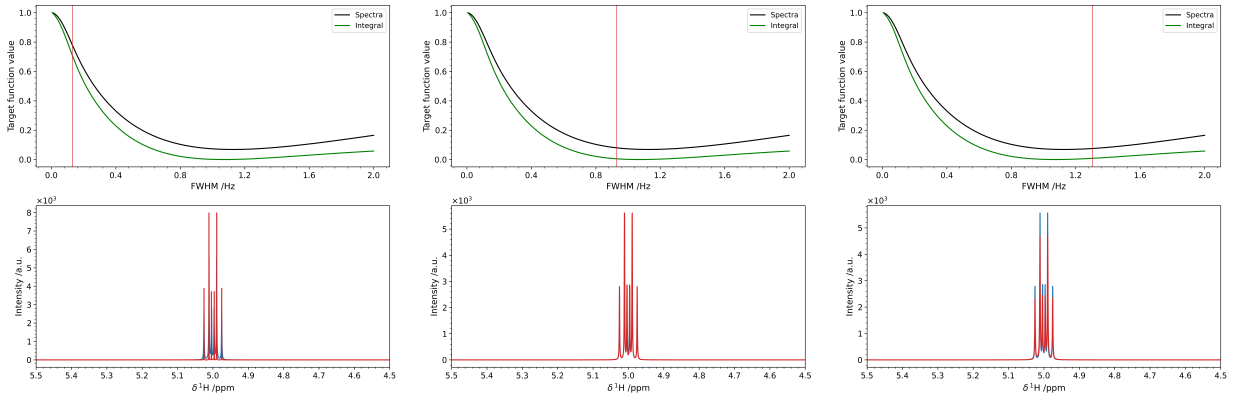

(a) Underestimated linewidth. (b) Actual linewidth. (c) Overestimated linewidth.

Figure S6: A signal centered at 5 ppm was simulated at 700 MHz ^{1}H Larmor frequency as a doublet of triplets with scalar coupling constants $J = 15$ and 10 Hz (blue trace, bottom panels). All the six features of the multiplet have the same linewidth of 1 Hz. The model signal (red trace, bottom panels) was simulated with the same parameter, and its FWHM is varied from 5×10^{-3} to 2 Hz in steps of 5×10^{-3} Hz. For each of these values, the value of the target function was computed on the difference of the spectra (equation 4, black trace in the top panels) and of their integrals (equation 5, green trace in the top panels). Here are shown three particular values of FWHM, marked with a red line on the top panels: one lower than the actual one (a), the true value (b), and a greater one (c). The figure shows that both target functions have only one very broad minimum, thus they are quite insensitive to variations of the linewidth.

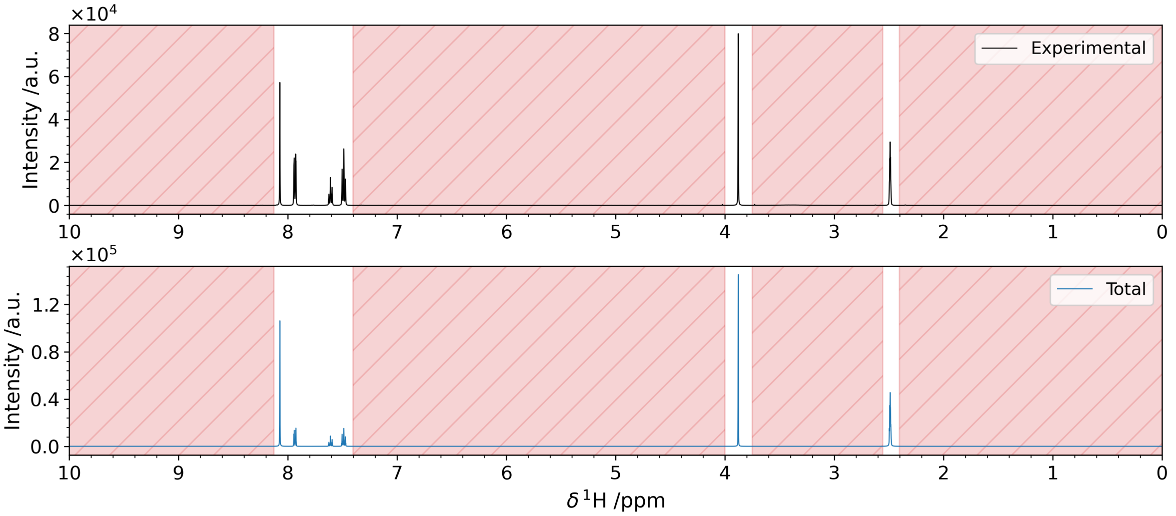

Figure S7: At each call of the target function, the simulated spectrum is computed on the whole spectral width according to equation 2. Then, the regions that do not contain relevant signals (red spans on the figure) are removed. The target value is thus the squared norm of the difference between experimental and model spectrum, only in the non-highlighted windows.

4 Additional data about pyIHM results

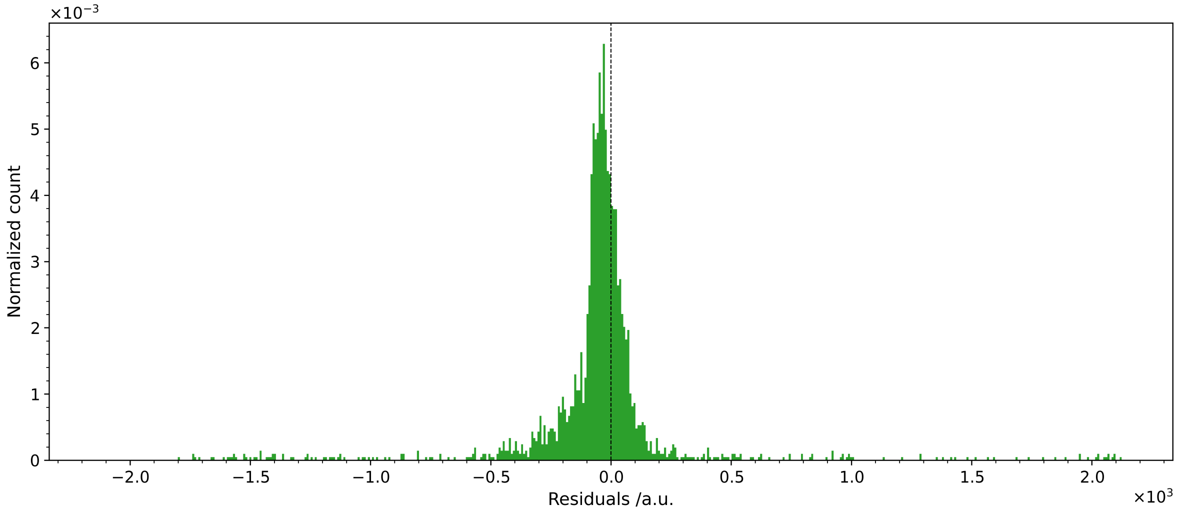

Figure S8: Histogram of the residuals of the the pyIHM deconvolution performed on a mixture of benzoic acid and dimethylterephtalate in DMSO (figure 2). The intensities were scaled to the integral of the total model trace for normalization purposes.

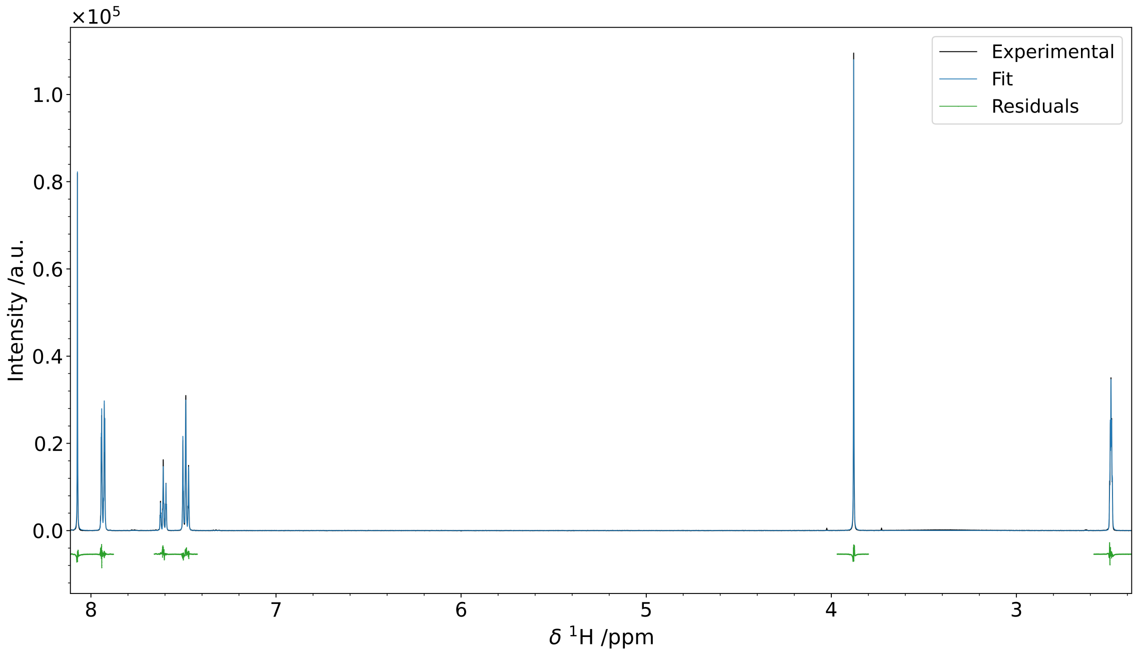

Figure S9: Result on the pyIHM deconvolution performed on a mixture of benzoic acid (blue) and DMTP (orange) in DMSO (red). The residuals of the fit are shown in green with a slight offset. Details on their statistical properties are given in figure S8. The "fast" optimization was used in this case. The intensities were scaled to the integral of the total model trace for normalization purposes. Table S2 compares the obtained composition with the one obtained by peak integration.

Table S2: Composition of the mixture of benzoic acid and dimethylterephtalate in DMSO computed with pyIHM (figure S9), compared with the intensities obtained by peak integration. The "fast" optimization was used in this case.

Component	from pyIHM	from integr.
BzAc	59.69%	60.23%
DMSO	19.83%	19.82%
DMTP	20.48%	19.95%

Table S3: Composition of the mixture of benzoic acid and ethyl carbonate in DMSO computed with pyIHM (figure S12), compared with the intensities obtained by peak integration. The "fast" optimization was used in this case.

Component	from pyIHM	from integr.
BzAc	48.57%	50.24%
DMSO	13.08%	12.75%
EC	38.35%	37.01%

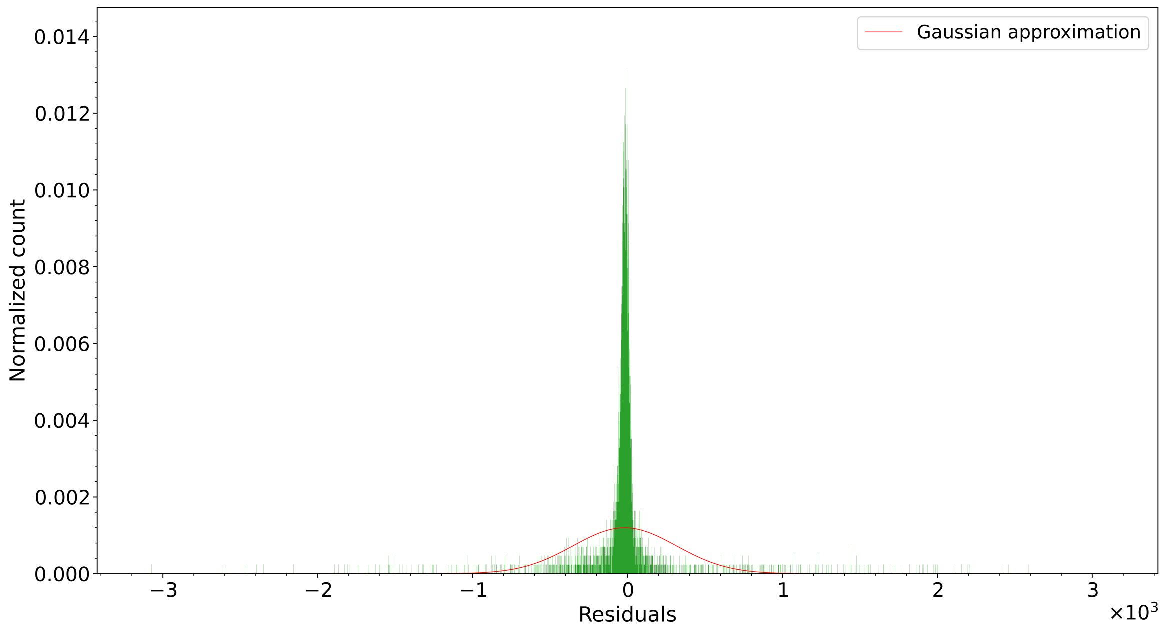

Figure S10: Histogram of the residuals of the the pyIHM deconvolution performed on a mixture of benzoic acid and dimethylterephtalate in DMSO (figure S9). The intensities were scaled to the integral of the total model trace for normalization purposes.

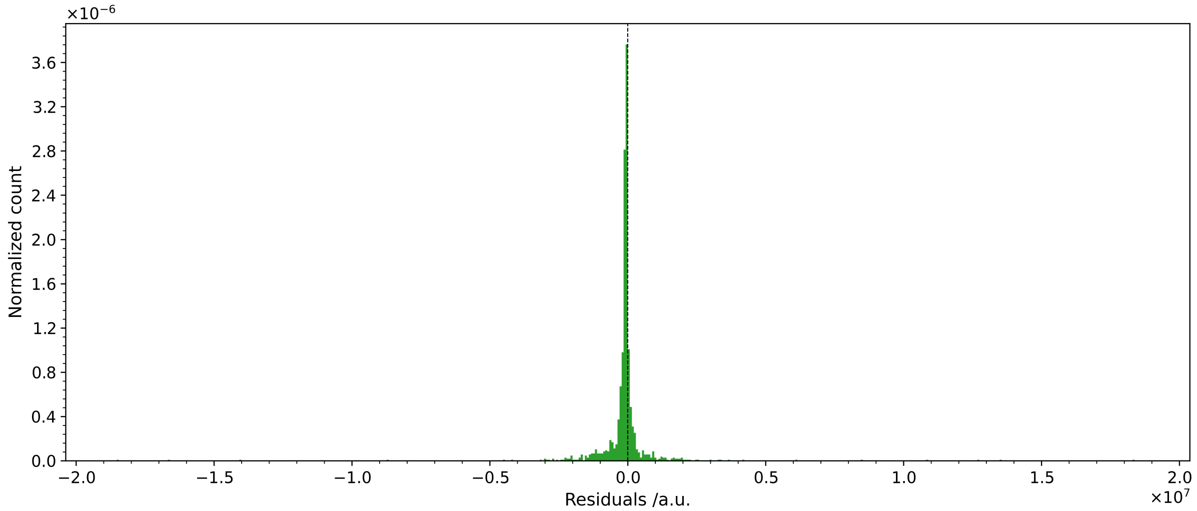

Figure S11: Histogram of the residuals of the the pyIHM deconvolution performed on a mixture of benzoic acid and ethyl carbonate in DMSO (figure 3). The intensities were scaled to the integral of the total model trace for normalization purposes.

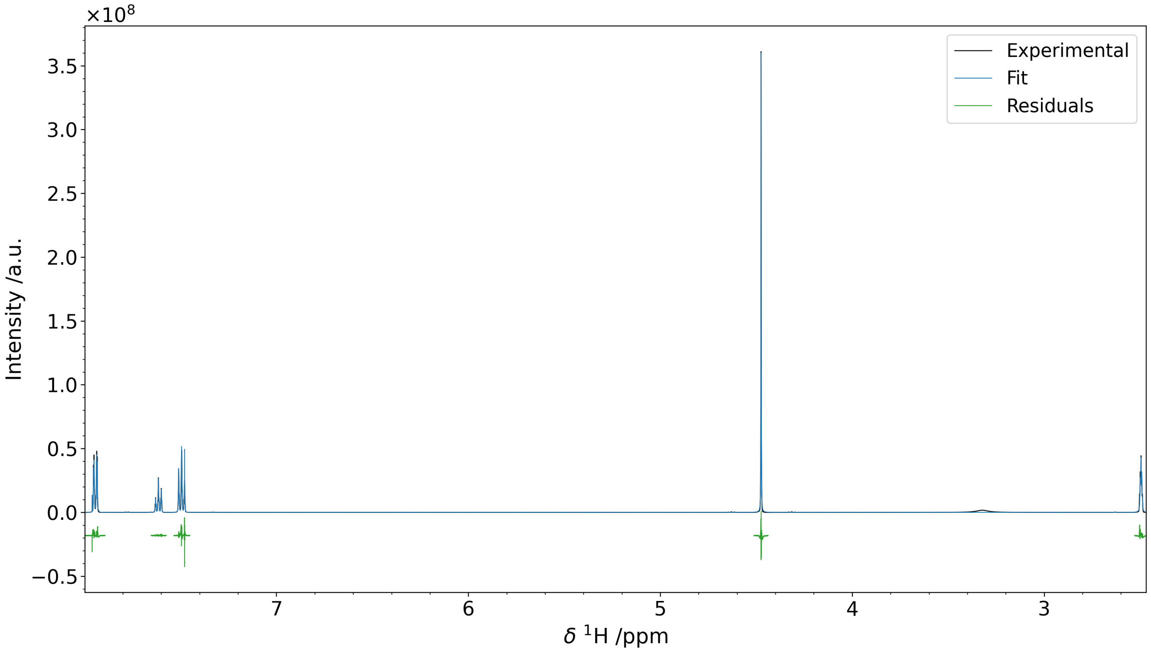

Figure S12: Result on the pyIHM deconvolution performed on a mixture of benzoic acid (blue) and ethyl carbonate (orange) in DMSO (red). The residuals of the fit are shown in green with a slight offset. Details on their statistical properties are given in figure S13. The "fast" optimization was used in this case. The intensities were scaled to the integral of the total model trace for normalization purposes. Table S3 compares the obtained composition with the one obtained by peak integration.

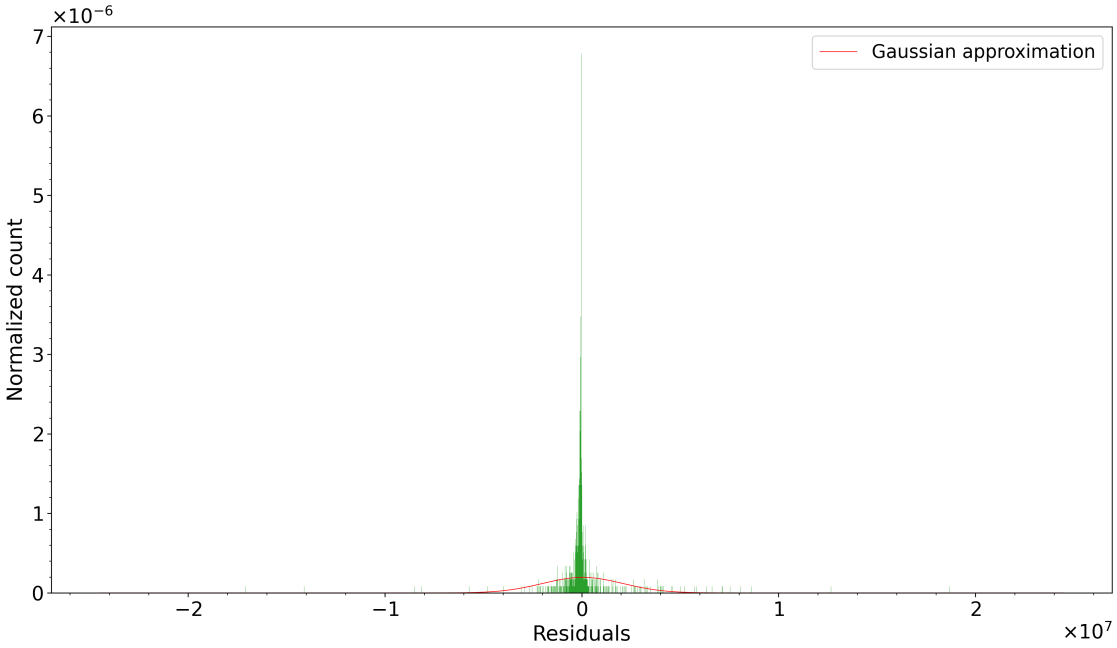

Figure S13: Histogram of the residuals of the the pyIHM deconvolution performed on a mixture of benzoic acid and ethyl carbonate in DMSO (figure S12). The "fast" optimization was used in this case. The intensities were scaled to the integral of the total model trace for normalization purposes.

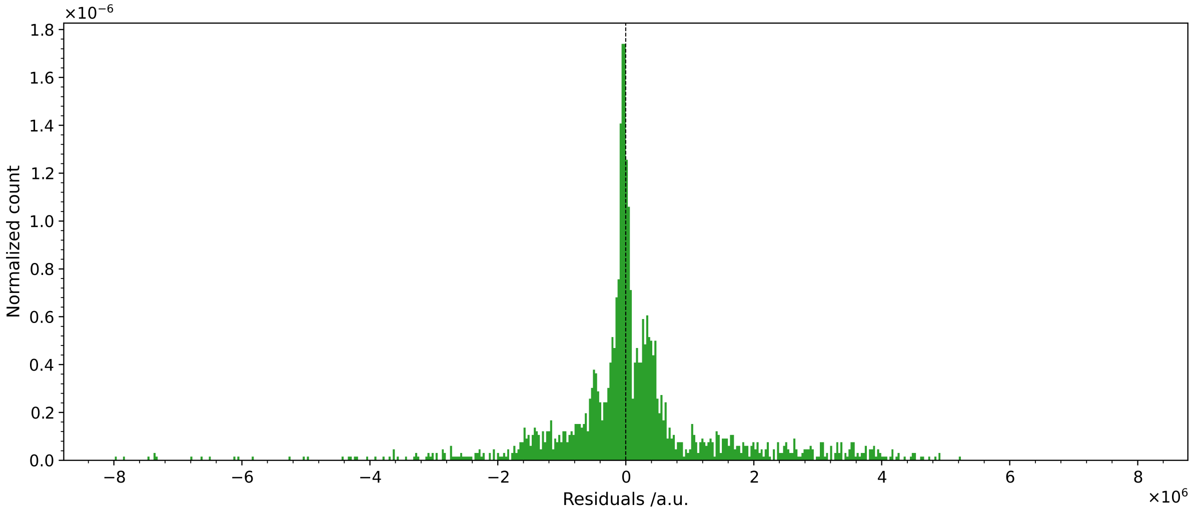

Figure S14: Histogram of the residuals of the the pyIHM deconvolution performed on a mixture of ochratoxin-a and tetrachloronitrobenzene (figure 4). The intensities were scaled to the integral of the total model trace for normalization purposes.

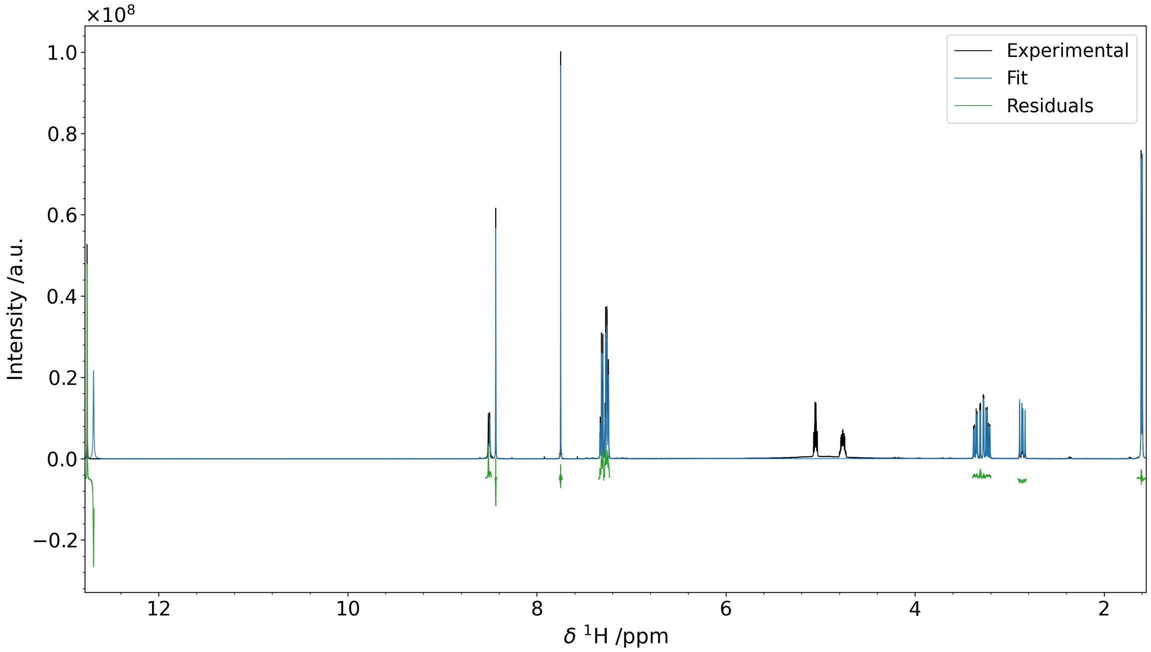

Figure S15: Result on the pyIHM deconvolution performed on a mixture of ochratoxin-a (blue) and TCNB (red). The residuals of the fit are shown in green with a slight offset. Details on their statistical properties are given in figure S16. The "fast" optimization was used in this case. The intensities were scaled to the integral of the total model trace for normalization purposes. The calculated purity with these results is 0.9684, to be compared with the 0.983 yielded by gravimetric analysis.

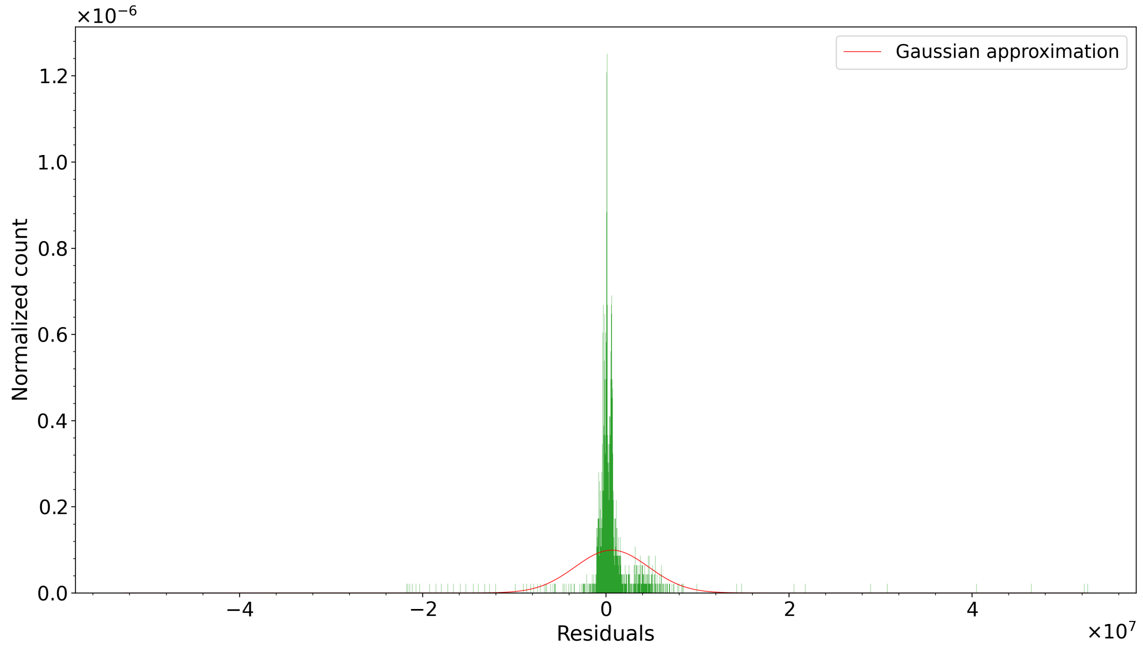

Figure S16: Histogram of the residuals of the the pyIHM deconvolution performed on a mixture of ochratoxin-a and tetrachloronitrobenzene (figure S15). The "fast" optimization was used in this case. The intensities were scaled to the integral of the total model trace for normalization purposes.

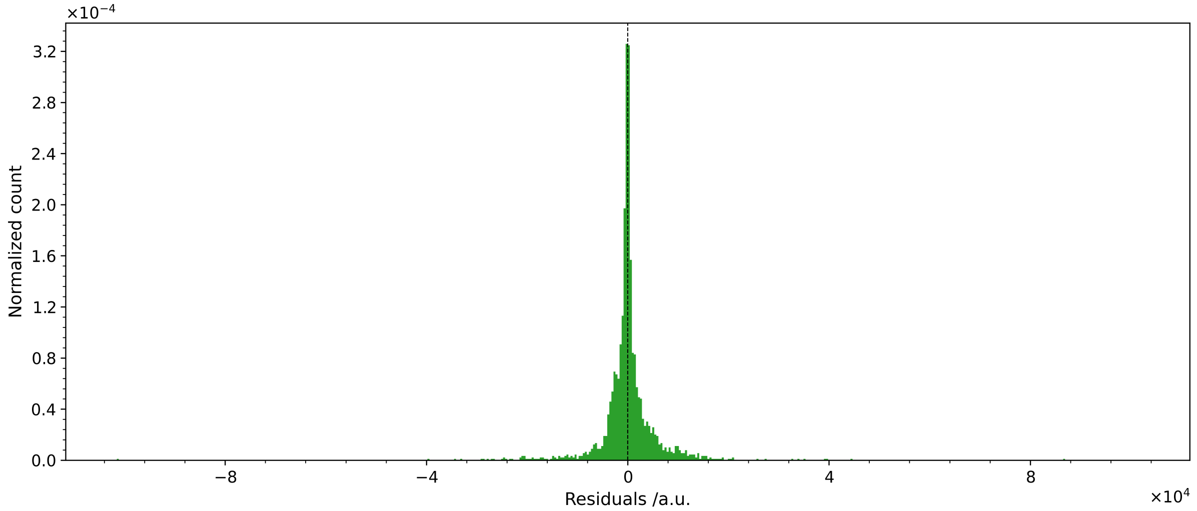

Figure S17: Histogram of the residuals of the the pyIHM deconvolution performed on a mock urine sample (figure 4). The intensities were scaled to the integral of the total model trace for normalization purposes.

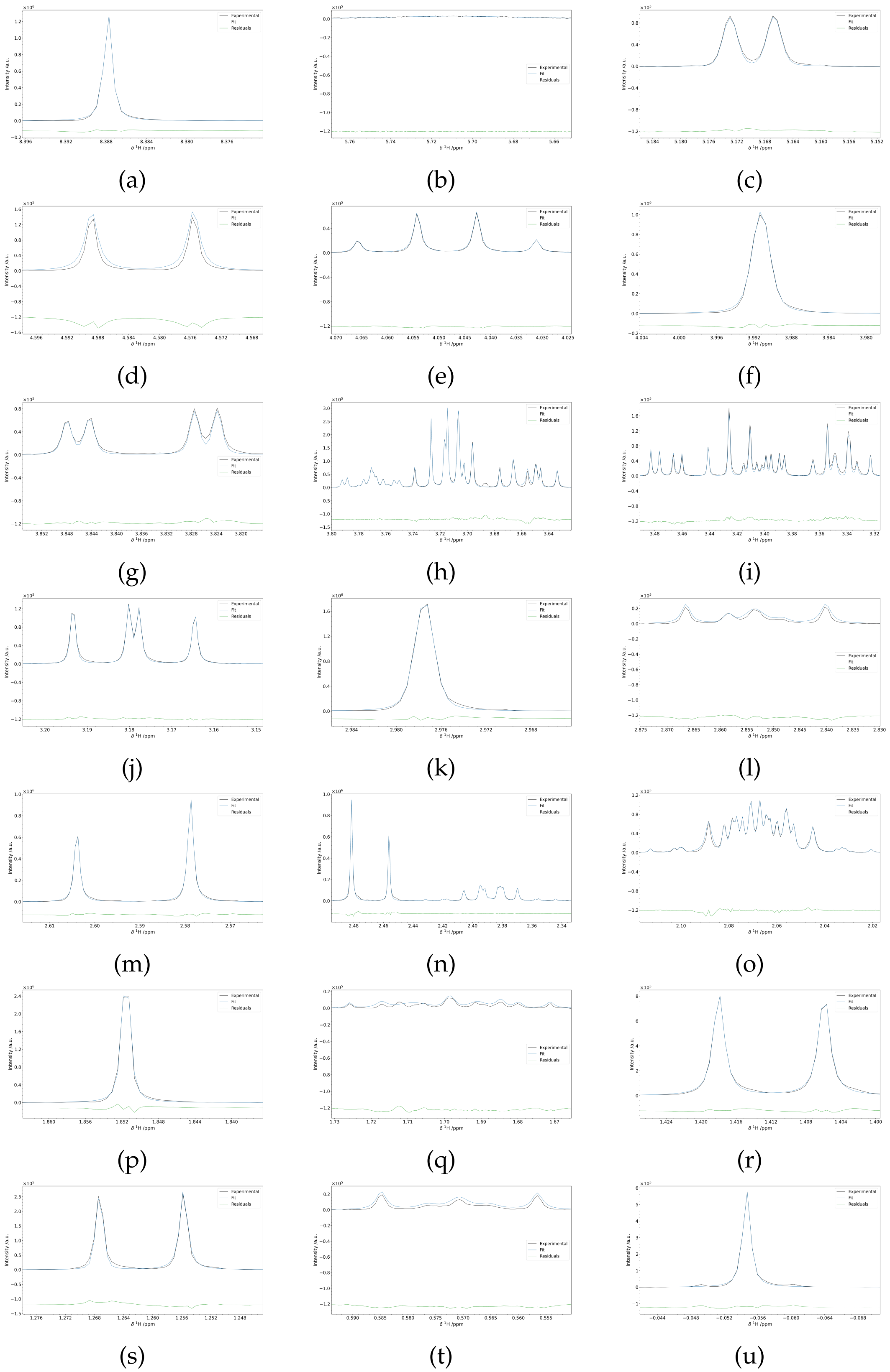

Figure S18: Details on the deconvolution of the mock urine sample performed in figure 5.
